# Prognostic Value of Red Blood Cell Distribution Width in Non-Cardiovascular Critically or Acutely Patients: A Systematic Review

**DOI:** 10.1371/journal.pone.0167000

**Published:** 2016-12-09

**Authors:** Rubin Luo, Jian Hu, Libing Jiang, Mao Zhang

**Affiliations:** Department of Emergency Medicine, Second Affiliated Hospital, School of Medicine& Institute of Emergency Medicine, Zhejiang University, Hangzhou, China; Azienda Ospedaliero Universitaria Careggi, ITALY

## Abstract

**Background:**

RDW (red cell distribution width) has been reported to been associated with the prognosis of patients with cardiovascular diseases. However, RDW is often overlooked by clinicians in treating patients with non-cardiovascular diseases, especially in an emergency. The objective of this systematic review is to explore the prognostic value of RDW in non-cardiovascular emergencies.

**Methods:**

PubMed, EMBASE, and the Cochrane Central Register of Controlled Trials were systematically searched from their inception to December 31, 2015. We included studies examining the relationship between RDW and mortality rate by adjusting important covariables in non-cardiovascular emergencies. All included studies were divided into three groups. Group A: general critically ill patients; Group B: patients with infectious disease; Group C: other conditions. We extracted each study’ characteristics, outcomes, covariables, and other items independently.

**Results:**

A total of 32 studies were eligible for inclusion in our meta-analysis. Six studies belonged to Group A, 9 studies belonged to Group B and 17 studies belonged to Group C. Among these included studies, RDW was assessed as a continuous variable (per 1% increase) in 16 studies, as a binary variable in 8 studies, and as a categorical variable in 8 studies. In addition, AUCs (area under the receiver operating characteristic curve) of RDW for predicting mortality were reported in 25 studies. All studies were published between 2011–2015. The qualities of included 32 studies were moderate or high.

**Conclusion:**

The present systematic review indicates that the increased RDW is significantly associated with a higher mortality rate in an non-cardiovascular emergency. The low cost and readily accessible of this laboratory variable may strengthen its usefulness in daily practice in the future.

## Introduction

Red blood cell distribution width (RDW) is a measure of erythrocyte size variability and calculated as the (standard deviation) SD in red blood cell (RBC) size divided by the mean corpuscular volume. RBC differ in size, whereas, this difference would get smaller during ageing [1]. In addition, any disorders result in the release of immature erythrocyte or shortening the lifespan of RBC would cause the change of RDW. RDW has traditionally been used for the diagnosis of different type of anemia [2]. In recent years, considerable attention were paid to the prognostic value of RDW [3–6]. In 2007, Michael Felker and his colleagues reported that RDW was a strong independent predictor of morbidity and mortality in chronic heart failure patients [6]. Subsequently, many other scholars found the similar association between RDW and various clinical conditions, including cardiovascular diseases, community-dwelling older adults and general in-hospital patients [3–8].

As we all know, an accurate risk stratification system is important in emergency department or intensive care unit [9, 10]. And continues efforts have been made to develop such a system. However, up to now, ideal prognostic models are still lacking. RDW is cost-effective and is routinely reported in the complete blood count (CBC) [9–18]. A growing body of evidence indicates the importance of RDW in predicting mortality rate in critically or acutely ill patients [19–33]. Nevertheless, the value of RDW has often been neglected by almost all clinicians in non-cardiovascular conditions. Thus, the aim of this systematic review is to assess the potential association between the RDW levels and mortality in non-cardiovascular emergencies.

## Materials and Methods

This systematical review was performed according to the Preferred Reporting Items for Systematic Reviews and Meta-Analyses (PRISMA, S1 Checklist) statement which was published in 2009 [34].

### Literature search and inclusion criteria

PubMed, EMBASE, and the Cochrane library were systematically searched from their inception to December 31, 2015. As RDW is not referenced by the Medical Subject Headings, it was used as a keyword to identify relevant studies only. The bibliographies of relevant reviews or meta-analysis were also screened to identify potential eligible studies.

The inclusion criteria: patients with a diagnosis of non-cardiovascular disease were included and those who were diagnosed with cardiovascular diseases, such as heart failure, myocardial infarction and so on were excluded. In addition, patients with malignant tumor were also excluded; Effect sizes [odds ratios (ORs) or hazard ratios (HRs) or AUC and their 95% confidence intervals (CIs)] were available; Randomized controlled study or observational study; The primary outcome was all-cause mortality.

### Data extraction and quality assessment

Data extraction was performed independently by two authors. The following data were extracted using a standard form: characteristics of each study (publication year, the first author, study design, the primary endpoint and the type of population), characteristics of all included patients (the mean age, male/female, and number of included patients), unadjusted and adjusted size effects (ORs or HRs or AUCs and their CIs) and important confounders (APACHEⅡ, age, hematocrit, hemoglobin, mean corpuscular volume, mean corpuscular hemoglobin, mean corpuscular hemoglobin concentration, C-reactive protein, sepsis, mechanical ventilation, admission type, leukocyte count and so on). Newcastle-Ottawa Scale was used to assess the methodological quality of included observational studies (http://www.ohri.ca/programs/clinical_epidemiology/oxford.asp). This scale consists of three domains: Selection, Comparability, and Outcome. Selection is evaluated through four items: representativeness of the exposed cohort, selection of the non-exposed cohort, ascertainment of exposure to implants and demonstration that outcome of interest was not present at start of study. Comparability is evaluated through one item: study controls for confounders. Outcome is evaluated through three items: assessment of outcome, duration of follow up and completeness of follow up. A study can be awarded with a maximum of one star for each numbered item within selection and outcome categories. A maximum of two stars can be given, as for comparability.

### Data analysis

RDW was reported in different forms [dichotomous variable (i.e. normal vs. abnormal); continuous variable (i.e. per 1% increase); and ordinal categorical variable (i.e. tertiles, quartiles)]. The AUC of RDW for mortality prediction was also regarded as an additional effect size. All included studies were divided into three groups: Group A: general critically ill patients; Group B: patients with infectious disease; Group C: other conditions. If applicable, the extracted effect sizes were used for quantitative analysis. ORs and HRs could be combined because of similar magnitude [35]. The method of “generic inverse variance” was applied in this model (http://www.cochrane.org/handbook). The data required for the generic inverse variance method are an estimate for the relative effect and its standard error (SE) for each of the studies. Each study is given a weight which is equal to the inverse of the variance of the effect estimate (i.e. one divided by the standard error squared). Then, we need to enter the napierian logarithm (ln) of the effect size and the standard error of the ln (effect size). If these two values are entered as the effect estimate and standard error, from them the software will calculate the effect size and 95%CI (this was not on the log scale). In our study, we needed to use the 95% CI to work backwards and calculated the SE of the ln (effect size). Heterogeneity between studies was assessed by chi-squared test and I^2^ statistic, and *P*<0.1 or I^2^ >50% indicated the presence of significant heterogeneity. Due to the wide clinical and methodological variability between studies, we chose random effects model to pool the results. Publication biases were assessed using Egger’s tests. All statistical analysis was performed on STATA 12.0 (SERIAL NO.40120519635), and RevMan 5.2.10 (http://tech.cochrane.org/revman/download).

## Results

### Search results

**Fig 1:**
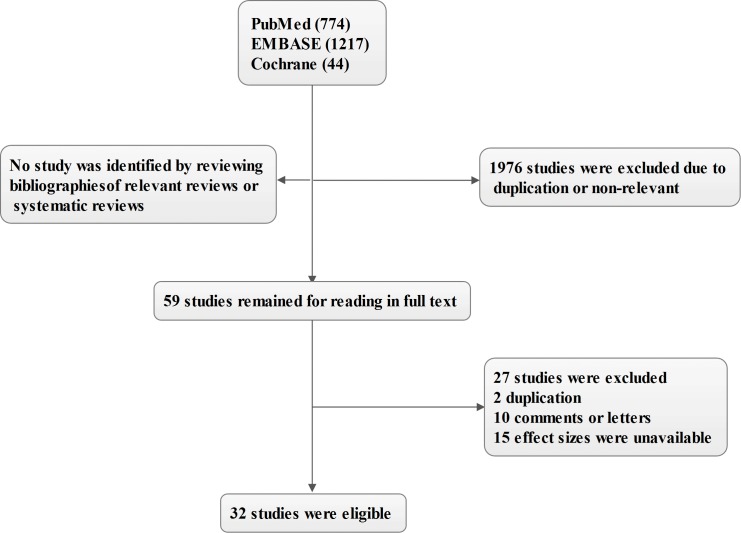
Flowchart of study selection.

A total of 32 observational studies met the pre-defined inclusion criteria and were included [1, 2, 9–11, 13–25, 27–29, 31–33, 36, 37]. The flow sheet of research selection was showed in Fig 1. There were no randomized controlled trials focusing on this topic were included. In addition, because of the studies by Purtle et al and Bazick et al were conducted in the same hospitals and derived from the same study periods [2, 12] and the risk of data duplication was inevitable, thus the study by Purtle et al was excluded [12]. A similar situation also occurred in the studies by Braun et al [26, 28], and his early study was excluded [26]. There was no additional studies were obtained through reviewing the bibliographies of relevant reviews or systematic reviews and there was no discrepancy between two reviewers.

### Study characteristics

The characteristics of all included studies were showed in Table 1 and Table 2. Among the 32 included studies, 6 studies belonged to the group A [1, 2, 9–11, 38], 9 studies belonged to the group B [23–25, 27–29, 31–33], and 17 studies belonged to the group C [13–22, 36, 37, 39–43]. As for the effect size, RDW was reported as a continuous variable (per 1% increase) in 16 studies [1, 9–11, 16, 17, 21–23, 29, 31, 32, 36, 37, 40, 43], as a binary variable in 8 studies [14, 16, 18, 19, 26, 33, 36, 40], and as a categorical variable in 8 studies [2, 13, 17, 22, 25, 27, 29, 42]. The AUC of RDW for mortality prediction was reported in 25 studies. However, the 95% CIs of AUCs were not reported in 6 studies [2, 16, 22, 24, 29, 36]. All studies were published between 2011 and 2015. The quality of the 32 studies included in this systematic review was listed in Table 3. The significant clinical heterogeneity between studies prevented us from combining the results of individual study, and a qualitative report was presented.

**Table 1:** Characteristics of Included Studies.

Study/Year	Study Design	Population	No. Total	Age, Yr	Male (%)	RDW Categorization	Outcomes Assessed	Type of Analysis
**General critically ill patients**								
Wang et al.[9] /2011	Prospective, single center	Adult ICU patients	602	Mean:70.39	58.1	Continuous (per 1% increase); Ordered Categorical (11.2 -13. 4%, 13.5-14.6%, 14.7-26.9%)	Primary: ICU mortality Secondary: length of hospital stay	Unadjusted and multivariable analysis
Bazick et al. [2] /2011	Retrospective, multiple center	Adult critically ill patients	51413	Mean:61.7	58.21	Ordered Categorical (≤13.3%, 13.3-14%, 14-14.7%, 14.7-15.8%, >15.8%)	Primary:30-day mortality Secondary: 90-day, 365- day, in-hospital mortality and blood stream infection	Unadjusted and multivariable analysis
Hunziker et al. [11] /2012	Retrospective, multiple center	Adult ICU patients	17922	Mean:63.2	56.9	Continuous (per 1% increase)	Primary: in-hospital mortality Secondary: ICU mortality and 1-year mortality	Unadjusted and multivariable analysis
Meynaar et al. [1]/2013	Retrospective, single center	Adult ICU patients	2915	Mean:65.1	57.15	Continuous (per 1% increase), Ordered Categorical (<43.2fl, 43.2-46.09fl, 46.1-49.69fl, >49.7fl	Primary: in-hospital mortality	Unadjusted and multivariable analysis
Zhang et al. [10] /2013	Retrospective, single center	Adult ICU patients	1539	Mean:61.8	65.35	Continuous (per 1% increase)	Primary: in-hospital mortality Secondary: ICU length of stay	Multivariable analysis
Hatice et al. 2015/[38]	Retrospective, single center	Adult ICU patients with community acquired intra- abdominal sepsis	103	Mean: 64	53.4	Continuous (per 1% increase)	Primary: hospital mortality	Unadjusted and multivariable analysis
**Patients with inflammation**								
Sadaka et al. [29] /2013	Retrospective, single center	Adult sepsis patients	279	Mean:67.4	51.4	Continuous (per 1% increase), Ordered Categorical (<13.5%, 13.5-15.5%, 15.6-17.5%, 17.6-19.4%, >19.4%)	Primary: in-hospital mortality Secondary: ICU mortality	Multivariable analysis
Jo et al. [25] /2013	Retrospective, single center	Adult sepsis and septic shock patients	566	Mean:70	55.5	Ordered Categorical(≤14, 14.1-15.7, ≥15.8)	Primary:28-day mortality Secondary: the rate of positive blood culture, renal replacement therapy, and admission to ICU	Multivariable Cox analysis
Tian et al. [23] /2014	Retrospective, single center	Adult septic shock patients	132	Mean of survivor: 69.28, Mean of nonsurvivor: 74.42	65.2	Continuous (per 1% increase)	Primary:28-day mortality	Multivariable analysis
Chen et al. [24] /2014	Retrospective, single center	Adult septic shock patients	219	Mean:69.89	59.4	Continuous (per 1% increase)	Primary:90-day mortality	Multivariable Cox analysis
Lee et al. [27] /2013	Retrospective, single center	Patients with CAP (age>18y)	744	Mean:70.1	32	Ordered Categorical (≤13.3%, 13.3-14.1%, 14.1-15.2%, >15.2%)	Primary:30-day mortality Secondary: hospital length of stay, use of vasopressors, ICU admission and mechanical ventilator requirement	Multivariable regression/Cox analysis
Braun et al. [28] /2014	Retrospective, single center	Patients with CAP (age>18y)	3815	Median:70	56.4	Binary (≤15% vs >15%)	Primary:90-day mortality Secondary: complicated hospitalization(defined as at least one of the following: In- hospital mortality, length of stay of at least 10 days or ICU admission)	Multivariable analysis
Ku et al. [31] /2012	Retrospective, single center	Patients with Gram- negative bacteremia	161	Mean:64.98	44.7	Binary (≤14.6% vs >14.6%) Continuous (per 1% increase)	Primary:28-day mortality	Unadjusted and multivariable Cox analysis
Seyhan et al. [32] /2013	Retrospective, single center	Patients diagnosed with COPD	270	Mean: 61	77	Binary (≤15.5% vs >15.5%) Continuous (per 1% increase)	Primary: mortality. Median follow-up was 36 months.	Unadjusted and multivariable analysis
Guray et al. [33] /2014	Retrospective, multiple center	Patients diagnosed with definite infective endocarditis	100	Mean: 47.8	61	Binary (≤15.3% vs >15.3%)	Primary:1-year mortality	Unadjusted and multivariable Cox analysis
**Other critically or acutely ill conditions**								
Kim et al. [13] /2012	Retrospective, single center	Out-of- hospital cardiac arrest victims	219		62.1	Ordered Categorical (≤13.1%, 13.2-14.%, 14.1-15.4%, >15.5%)	Primary:30-day mortality	Unadjusted and multivariable cox analysis
Oh et al. [16] /2012	Retrospective, single center	Patients with AKI who were treated with CRRT	470	Mean of RDW<14.6% 62.8; RDW≥14.6%, 61.5	RDW<14.6%, 66; RDW≥14.6%, 63.7	Binary (<14.6% vs ≥14.6%) Continuous (per 1% increase)	Primary:28-day mortality	Unadjusted and multivariable cox analysis
Hong et al. [17] /2012	Retrospective, single center	Patients with acute dyspnea who visited the ED	907		54	Continuous (per 1% increase), Ordered Categorical (<12.9%, 12.9-14.3%, >14.3%)	Primary:30-day mortality Secondary: composite of 30- day mortality or re-hospitalization	Unadjusted and multivariable cox analysis
Senol et al. [15] /2013	Retrospective, single center	Patients with acute pancreatitis	102	Median:56.5	42.2	Continuous (per 1% increase)	Primary: in-hospital mortality	Unadjusted and multivariable analysis
Majercik et al. [22] /2013	Retrospective, multiple center	Adult trauma patients	9538	Mean: 48	62.1	Continuous (per 1% increase), Ordered Categorical (11.3- 13.0%, 13.1-13.5%, 13.6- 14.0%, 14.1-14.9%, 15.0- 32.4%)	Primary:30-day mortality Secondary:1-year mortality (from 31 to 365 days of follow -up)	Multivariable Cox analysis
Garbharran et al. [21] /2014	Prospective, single center	Hip fracture cases	698	Mean: 78	33	Continuous (per 1% increase), Ordered categorical(10-13%, 13.1-14.1%, 14.2-15.2%, >15.3)	Primary: in-hospital mortality, 120-day mortality and 1-year mortality	Multivariable Cox analysis
Bilgic et al. [20] /2014	Retrospective, single center	Patients with acute mesenteric ischemia	61	Median: 72	59	Continuous (per 1% increase)	Primary: in-hospital mortality	ROC analysis
Kang et al. [14] /2014	Retrospective, single center	Patients after organophos phate insecticide poisoning	102	Mean:57.5	66.7	Binary (≤13.5% vs >13.5%)	Primary: 30-day mortality	Unadjusted and multivariable cox analysis
Zorlu et al. [18] /2012	Prospective, multiple center	Patients with acute pulmonary embolism	136	Mean:63	48	Binary (≤14.6% vs >14.6%)	Primary: in-hospital mortality	Unadjusted and multivariable cox analysis
Sen et al. [19] /2014	Retrospective, single center	Patients with acute pulmonary embolism	208	Mean:57.87	38	Binary (≤16.25% vs >16.25%)	Primary:100-day mortality	Unadjusted and multivariable analysis
Ozsu et al. [37] /2014	Retrospective, single center	Patients with acute pulmonary embolism	702	Median:68	35	Continuous (per 1% increase), Ordered categorical(≤13.6%, 13.7-14.5%, 14.6-16.3%, >16.3%)	Primary: in-hospital mortality	Unadjusted and multivariable analysis
Mucsi et al. [36] /2014	Prospective, Single center	Prevalent kidney transplant recipients	723	Mean:51	50	Continuous (per 1% increase), Binary: (≤13.7% vs >13.7%)	Primary: all -cause mortality	Unadjusted and multivariable cox analysis
Miriam et al. 2015/[40]	Retrospective, single center	Adult internal medicine ward patients	586	Mean: 62.3	46.6	Binary (≤14.7% vs >14.7%); Continuous (per 1% increase)	Primary: 60-month mortality	Multivariable analysis
Yao et al. 2014/[41]	Retrospective, single center	Patients with acute pancreatitis	106	MAP:48.2 SAP:60.5	49	Ordered categorical (>13.3%, 12.6-13.3%, <12.6%)	Primary: 3-month mortality	Unadjusted
Lv et al. 2015/[42]	Prospective, single center	Hip fracture cases	1479	Median: 73	41.6	Ordered categorical (< 12.50%,12.51%-13.10%,13.11%- 13.80%, >13.81%)	Primary: 2- year mortality	Unadjusted and multivariable analysis
Dinc et al. 2015/[43]	Retrospective, single center	Patients with acute mesenteric ischemia	73	Mean: 69.3	57.53	Continuous (per 1% increase),	Primary: post-operative mortality	Multivariable analysis
Wang et al. 2015/[39]	Retrospective, single center	Patients with acute pancreatitis	120	Mean: 51.2	41.7	Ordered categorical (>13.4%, 12.6%-13.4%,≤12.6%)	Primary: 3-month mortality	None

RDW = red cell distribution width, ROC = receiver operating characteristic.

**Table 2:** The Effect of RDW on Mortality.

Study/Year	Mortality	Univariable Analysis or Multivariable Analysis		Adjusted Variables	Notes
**General critically ill patients**					
Wang et al. [9]/2011	ICU: 21.1% Within categories: 11.2-13.4: 11.2%, 13.5-14.6: 18.8%, 14.7-26.9: 33.8%	Univariable Analysis ICU mortality OR: 1.784 (1.475-2.158) per 1% increase	Multivariable Analysis ICU mortality OR: 1.551 (1.25-1.926) per 1% increase	Age, APACHE-II, CRP, GFR, hemoglobin, albumin	ICU mortality AUC:0.672 (0.645-0.699) Cut-off: 14.8% Sensitivity: 51.2% Specificity: 74.7%
Bazick et al. [2] /2011	30-day: 14.15% 90-day: 18.67% 365-day: 26.27% In-hospital: 12.80%	Univariable Analysis 30-day mortality ≤13.3%, OR: 1 13.3-14%,OR: 1.52(1.4-1.66) 14-14.7%,OR: 1.91(1.74-2.1) 14.7-15.8%, OR: 2.84(2.6- 3.11) >15.8%, OR: 5.02(4.64-5.44) Bloodstream infection ≤13.3%, OR:1 13.3–14%, OR: 1.30 (1.13– 1.50) 14–14.7%, OR: 1.56 (1.35– 1.81) 14.7–15.8%,OR: 1.75 (1.52– 2.01) >15.8%, OR: 1.96 (1.73– 2.23)	Multivariable Analysis 30-day mortality ≤13.3%, OR: 1 13.3-14%, OR: 1.19(1.08-1.3) 14-14.7%,OR:1.28(1.16-1.42) 14.7-15.8%, OR: 1.69(1.53- 1.86) >15.8%, OR: 2.61(2.23-2.86) Bloodstream infection ≤13.3%, OR: 1 13.3–14%, OR: 1.19 (1.03– 1.38) 14–14.7%, OR: 1.34(1.15– 1.56) 14.7–15.8%, OR: 1.40(1.20– 1.63) >15.8%, OR: 1.44(1.24–1.66)	Age, sex, race, patient type, Charlson index, Cr, Hct, WBC, BUN, MCV, sepsis, AMI, transfusion, CHF, CABG	30-day mortality: AUC:0.67 (CI not given) Bloodstream infection: AUC: 0.57 (CI not given)
Hunziker et al. [11] /2012	In-hospital: 11.2% ICU: 7.6%	Univariable Analysis In-hospital mortality OR: 1.2 (1.16-1.24) per 1% increase ICU mortality OR: 1.18 (1.14-1.22) per 1% increase	Multivariable Analysis In-hospital mortality OR: 1.14 (1.08-1.19) per 1% increase ICU mortality OR: 1.10 (1.06-1.15) per 1% increase	SAPS, age, gender, Hct, and different comorbidities	The multivariable adjusted OR for 1-year mortality was 1.20 (1.14–1.26) per 1% increase 1-year mortality: AUC: 0.73 (0.72- 0.74)
Meynaaret al. [1]/2013	In-hospital: 13.3% Within categories: <43.2fl: 7.4% 43.2-46.09fl: 10.9% 46.1-49.69fl: 11.5% >49.7fl: 23%	Univariable Analysis In-hospital mortality <43.2fl,OR:1 43.2-46.09fl, OR: 1.53 (1.06- 2.19) 46.1-49.69fl, OR: 1.62 (1.13- 2.34) >49.7fl, OR: 3.73 (2.79-5.18)	Multivariable Analysis In-hospital mortality OR: 1.04 (1.02-1.06) per 1% increase	APACHEⅡ, age, Hct, Hb, MCV, MCH, MCHC, CRP, sepsis, mechanical ventilation, admission type, leukocyte count	None
Zhang et al. [10] /2013	In-hospital: 29.6%	None	Multivariable Analysis In-hospital mortality: OR, 1.11 (1.04-1.18) per 1% increase	Age, gender, RRT, albumin, CRP, Hb, Charlson index	In-hospital mortality AUC:0.6202(0.589- 0.651) Patients with higher RDW will have longer LOS in ICU. Changes in RDW during a short follow up period were not associated with mortality
Hatice et al. 2015/[38]	In-hospital: 50%	None	Multivare Analysis In-hospital: for male HR, 1.07 (0.92–1.25); for female HR, 1.20 (0.95–1.54) per 1% increase	Age, source of sepsis, comorbidity, WBC, CRP, RDW, APACHE II and length of hospital stay	In-hospital mortality AUC: 0.867 (0.791-0.942) Cut-off 16% Sensitivity: 94.23% Specificity: 76.47%
**Patients with inflammation**					
Sadaka et al. [29]/2013	In-hospital: 47.1% ICU: 33.6%	None	Multivariable Analysis In-hospital mortality: OR, 1.27 (1.11-1.46) per 1% increase <13.5%, OR:1 13.5-15.5%, OR: 4.6 (1.0- 23.4) 15.6-17.5%, OR: 8.0 (1.5- 41.6) 17.6-19.4%, OR: 25.3 (4.3- 149.2) >19.4% OR:12.3 (2.1-73.3) ICU mortality: OR, 1.29 (1.13-1.48) per 1% increase <13.5%, OR:1 13.5-15.5%, OR: 4.6 (0.6- 38.9) 15.6-17.5%, OR: 9.1 (1.1- 78.2) 17.6-19.4%, OR: 20.4 (2.3- 183.4) >19.4% OR: 18.8 (2.0-178.2)	Age, gender, BMI,APACHE II score, SOFA score, comorbidities, and number of organ failures	In-hospital mortality:AUC: 0.74 (CI not given). The AUC was 0.69 for APACHE II and 0.69 for SOFA. When adding RDW to APACHE II, the AUC increased from 0.69 to 0.77
Jo et al. [25] /2013	28-day:29% Within categories: ≤14: 13.1% 14.1-15.7: 30.1% ≥15.8: 44.9%	None	Multivariable Analysis 28-day mortality: ≤14, HR:1 14.1-15.7, HR: 1.66(1-2.76) ≥15.8, HR: 2.57(1.53-4.34)	Age, gender, APACHE II score, albumin, cholesterol, Creatine, BUN, potassium, MCH, MCV, Hct, WBC, PH, MAP, liver disease, infection site	28-day mortality: AUC: 0.678(0.631- 0.724). The rates of renal replacement therapy, mechanical ventilation, and admission to ICU were not different across RDW tertiles
Tian et al. [23] /2014	28-day:50.8%	None	Multivariable Analysis 28-day mortality: OR, 1.402 (1.01-1.947) per 1% increase	SOFA score, APACHE II score, WBC, Hct, MCV, Hb	28-day mortality: AUC: 0.632(0.535- 0.729). Changes in RDW during a short follow up period were associated with mortality
Chen et al. [24] /2014	90-day:52.1% Within categories: ≤15: 38.9% >15: 77.3% 30-day:13.4%	None	Multivariable Analysis 90-day mortality: HR,1.122 (CI not given)	SOFA score, APACHE II score	90-day mortality: AUC: 0.723(CI not given)
Lee et al. [27] /2013	Within categories: ≤13.3%: 5.6% 13.3-14.1%: 7% 14.1-15.2%: 12.6% >15.2%: 28.5%	None	Multivariable Analysis 30-day mortality: ≤13.3%, OR:1 13.3-14.1%, OR: 0.73 (0.28- 1.91) 14.1-15.2%, OR: 1.11 (0.47- 2.62) >15.2% OR: 2.37 (1.04-5.42)	CURB-65, albumin, cholesterol, prothrombin time	Secondary outcomes, also differed significantly among the RDW quartiles
Braun et al. [28] /2014	In-hospital: 14.3% 90-day: 24.6%	Univariable Analysis 90-day mortality: >15%, OR: 3.04(2.61-3.54) Complicated admissions: >15%, OR: 2.1(1.88-2.44)	Multivariable Analysis 90-day mortality: >15%, OR: 2.1(1.8-2.5) Complicated admissions: >15%, OR: 1.5(1.3-1.8)	Age, Na, SBP, HR, SaO_2_, WBC, Hb, BUN, Charlson index	None
Ku et al. [31] /2012	28-day:14.3% Within categories: ≤14.6%: 4.4% >14.6%: 27.1%	Univariable Analysis 28-day mortality: HR: 1.194 (1.073-1.329) per 1% increase	Multivariable Analysis 28-day mortality: HR: 1.174 (1.011-1.365) per 1% increase	Age, BUN, SOFA, Charlson index	28-day mortality: AUC: 0.764(0.65- 0.879).
Seyhan et al. [32] /2013	Within categories: ≤15.5%: 23% >15.5%: 78%	Univariable Analysis OR: 1.2 (1.15-1.3) per 1% increase	Multivariable Analysis OR: 1.12 (1.01-1.24) per 1% increase	Age, CVD, FEV_1_, PaCO_2_, albumin, anemia, CRP, PAH, RVD	RDW was positively correlated with CRP (r=0.21), RVD (r = 0.25), and PAH (r = 0.14)
Guray et al. [33] /2014	1-year:41% Within categories: ≤15.3%: 14% >15.3%: 56%	Univariable Analysis 1-year mortality: RDW>15.3%,HR:5.63(2.2- 14.38)	Multivariable Analysis 1-year mortality: RDW>15.3%,HR:3.07(1.06- 8.86)	Heart failure, renal failure, cardiac abscess, severe valvular regurgitation and presence of dehiscence	1-year mortality AUC: 0.7(0.59-0.8) Cut-off: 15.3% Sensitivity: 88% Specificity: 53%
**Other critically or acutely patients**					
Kim et al. [13] /2012	30-day:73.1% Within categories: ≤13.1%: 60% 13.2-14.%: 71.2% 14.1-15.4%: 72.7% >15.5%: 88.2%	Univariable Analysis 30-day mortality: ≤13.1%,HR:1 13.2-14.%, HR: 1.32 (0.83– 2.11) 14.1-15.4%, HR: 1.42 (0.89– 2.29) >15.5%, HR: 2.21 (1.38– 3.52)	Multivariable Analysis 30-day mortality: ≤13.1%,HR:1 13.2-14.%, HR: 1.25 (0.76– 2.06) 14.1-15.4%, HR: 1.29 (0.76– 2.19) >15.5%, HR: 1.95 (1.05–3.60)	Age, gender, initial shockable rhythm, Hct, platelet, BUN, Cr, albumin	30-day mortality: AUC: 0.61(0.53- 0.69)
Oh et al. [16] /2012	28-day:62.8%	Univariable Analysis 28-day mortality: ≥14.6,HR: 1.29(1.02–1.62) HR: 1.07 (1.02–1.12) per 1% increase	Multivariable Analysis 28-day mortality: ≥14.6,HR: 1.21 (1.01–1.71) HR: 1.06 (1.01–1.17) per 1% increase	Age, gender, CRP, Hb, albumin, total cholesterol, MAP, SOFA score	28-day mortality: AUC: 0.586(CI not given)
Hong et al. [17] /2012	30-day: 9.5% Within categories: <12.9%: 1.4% 12.9-14.3%: 8.5% >14.3%: 18.3%	Univariable Analysis 30-day mortality: HR: 1.33 (1.25–1.42) per 1% increase	Multivariable Analysis 30-day mortality: <12.9%, HR:1 12.9-14.3%, HR: 3.52 (1.20– 10.28) >14.3%, HR: 7.207, (2.49– 21.23) HR: 1.23 (1.12–1.34) per 1% increase	Charlson index, HR, previous use of angiotensin inhibitors, presence of pulmonary edema, hemoglobin, MCV, WBC, serum sodium, and albumin	30-day mortality: AUC: 0.746(0.7- 0.796) Cut-off: 14.5% Sensitivity: 66.3% Specificity: 70.2%
Senol et al. [15] /2013	In-hospital:13%	Univariable Analysis In-hospital mortality P=0.000(OR and its CI not given)	Multivariable Analysis In-hospital mortality P=0.001(OR and its CI not given)	Age, BUN, platelet, WBC, albumin, calcium	In-hospital mortality: AUC: 0.817(0.689- 0.946) Cut-off: 14.8%
Majercik et al. [22] /2013	30-day: Within categories: 11.3-13.0%: 2.2%,3.4% 13.1-13.5%: 1.8%,1.9% 13.6-14%: 3.6%, 3% 14.1-14.9%: 4.8%,3.9% 15-32.4%: 10%,6.2%	None	Multivariable Analysis 30-day mortality Male: HR: 1.17 (1.04-1.3) per quintile. 11.3-13.5%, HR: 1; 13.6-14%, HR: 1.41 (p = 0.10); 14.1- 14.9%, HR: 1.54 (p = 0.039); 15.0-32.4%, HR: 2.09 (p<0.001) HR, 1.19 (1.12-1.26) per 1% increase Female: HR: 1.17 (1.01-1.35) per quintile. 11.3-13.5%,HR: 1; 13.6-14%, HR: 1.06 (p = 0.85); 14.1- 14.9%, HR: 1.01 (p = 0.96); 15.0-32.4%, HR: 2.38 (p<0.001) HR, 1.08 (1.01-1.16) per 1% increase	Age, injury severity score, LOS, type of trauma, and each of the other complete blood cell count parameters	30-day mortality: AUC: 0.705 in males and 0.625 in females
Majercik et al. [22] /2013	1-year: Within categories: 11.3-13.0%: 0.5%,0.5% 13.1-13.5%: 0.4%,2.1% 13.6-14%: 0.8%,3% 14.1-14.9%: 1.7%,4.2% 15-32.4%: 8.3%,8.8%	None	Multivariable Analysis 1-year mortality: Male: HR: 1.52 (1.24-1.88) per quintile. 11.3-13.5%,HR: 1; 13.6-14%, HR: 1.05 (p = 0.92); 14.1- 14.9%, HR: 1.28 (p = 0.52); 15.0-32.4%, HR: 3.82 (p<0.001) HR, 1.27 (1.2-1.35) per 1% increase Female: HR: 1.43 (1.21-1.69) per quintile. 11.3-13.5%, HR: 1; 13.6-14%, HR: 1.45 (p = 0.27); 14.1- 14.9%, HR: 1.72 (p = 0.09); 15.0-32.4%, HR: 2.94 (p<0.001) HR, 1.22 (1.17-1.28) per 1% increase	Age, injury severity score, LOS, type of trauma, and each of the other complete blood cell count parameters	1-year mortality AUC: 0.820 in males and 0.723 in females
Garbharran et al. [21] /2014	1-year: 23% Within categories: 10-13%: 12% 13.1-14.1%: 15% 14.2-15.2%: 29% >15.3%: 36%	None	Multivariable Analysis In-hospital morality: HR:1.119(1-1.253) per 1% increase 120-day mortality: HR:1.134(1.047-1.227) per 1% increase 1-year mortality: HR:1.131(1.067-1.199) per 1% increase	Hb, MCV, age, gender, pre- fracture residence, required aid to mobilise indoors pre-fracture, ASA, Charlson index, post-operative delirium, cardiac, respiratory GI complicationsand serum creatinine	After excluding anaemic patients: In-hospital morality HR:1.21(1.04-1.41) per 1% increase 120-day mortality HR:1.17(1.04-1.31) per 1% increase 1-year mortality HR:1.27(1.16-1.4) per 1% increase
Bilgic et al. [20] /2014	In-hospital:57.4%	None	None	None	In-hospital mortality: AUC: 0.713(0.584- 0.841) Cut-off: 14.85% Sensitivity: 68.42% Specificity: 53.85%
Kang et al. [14] /2014	30-day: 20.6% Within categories: ≤13.5%: 1.7% >13.5: 48%	Univariable Analysis 30-day mortality: >13.5, HR: 4.76(2-11.33)	Multivariable Analysis 30-day mortality: >13.5, HR: 2.64(1.05-6.6)	Age, SBP, Hct, Cr, albumin unresponsive in AVPU scale	30-day mortality: AUC:0.675(0.522- 0.829) Cut-off: 13.5% Sensitivity: 57.1% Specificity: 84%
Zorlu et al. [18] /2012	In-hospital: 15.4% Within categories: ≤14.6%: 1.6% >14.6%: 27%	Univariable Analysis In-hospital mortality: >14.6%, HR: 19.789 (2.654– 147.5)	Multivariable Analysis In-hospital mortality: >14.6%, HR: 15.465 (1.811– 132.064)	Age, presence of shock, heart rate, oxygen saturation, Cr	In-hospital mortality: AUC: 0.734(0.646- 0.822) Cut-off: 14.6% Sensitivity: 95.2% Specificity: 53%
Sen et al. [19] /2014	100-day:14.42%	Univariable Analysis 100-day mortality: >16.25%, OR: 6.55 (2.153– 19.975)	Multivariable Analysis 100-day mortality: >16.25%, OR: 4.06 (1.229– 13.335)	Neutrophil/ lymphocyte ratio, platelet distribution width, sPESI, oxygen saturation, and CRP	100-day mortality: AUC: 0.646(0.557- 0.736) Cut-off: 16.25% Sensitivity: 79.2% Specificity: 55.6%
Ozsu et al. [37] /2014	In-hospital:12% Within categories: ≤13.6%: 5.8% 13.7-14.5%: 9.7% 14.6-16.3%: 13.1% >16.3%: 20%	Univariable Analysis In-hospital mortality: OR:1.2(1.1-1.3) per 1% increase	Multivariable Analysis In-hospital mortality: OR:1.2 (1.1-1.4) per 1% increase	Sex, RR, sPESI, CRP, D-dimer, MCV, and serum Tn-T, oxygen saturation	In-hospital mortality: AUC:0.649(0.584- 0.715) Cut-off: 15% Sensitivity:66% Specificity:59%
Mucsi et al. [36] /2014	Mortality:11% Median follow up time 35 months	Univariable Analysis HR:1.63(1.41–1.89) per 1% increase >13.7, HR: 2.74 (1.68–4.48)	Multivariable Analysis HR:1.60 (1.27–2.02) per 1% increase >13.7,HR: 1.33 0.76–2.35	age, gender, GFR, iron markers inflammatory markers, Charlson index, total time in ESRD,steroid use, mammalian target of rapamycin use, ACEi or ARB use	AUC:0.689(CI not given) Cut-off: 14% Sensitivity:63% Specificity:65%
Miriam et al. 2015/ [40]	60-month: 30% Within categories: ≤14.7: 39.8% >14.7: 17.5%	None	Multivariable Analysis 60-month mortality: >14.7, RR: 1.53 (1.11-2.11) Continuous, RR:1.21(1.13- 1.32)	Age, anemia, renal dysfunction, diabetes mellitus, coronary artery disease, chronic lung disease, heart failure, history of malignancy, complex nursing care and mechanical ventilation	None
Yao et al. 2014/[41]	3-month: 7.5%	None	None	None	3-month mortality AUC: 0.846(0.727- 0.964) Cut-off: 14.2% Sensitivity: 75% Specificity: 89.8%
Lv et al. 2015/[42]	2-year: 12.9% Within categories: < 12.50%: 7.2% 12.51%-13.1%: 10.9% 13.11%-13.8%: 12.2%, >13.81%: 22.2%	None	Multivariable Analysis 2-year mortality: RDW was examined as quartiles, HR 1.224 (1.057- 1.417) <12.50%, 1; 12.51%-13.10% 1.17 (0.71-1.91); 13.11%- 13.80% 1.14(0.70-1.86); >13.81% 1.83(1.14-2.93)	age, prior-myocardial infarction, chronic renal failure, ASA score, treatment, in-hospital pneumonia, in-hospital circulatory complications	None
Dinc et al. 2015/[43]	Post-operative Mortality: 54.8%		Multivariable Analysis Post-operative mortality: OR, 1.5115 (1.3287-6.8084)	age, gender, comorbid diseases, medications, blood biochemistry, complete blood cell count, pathology results and type of surgery	None
Wang et al. 2015/[39]	3-month: 50%	None		None	3-month mortality AUC: 0.894 (0.823-0.966) Cut-off: 14.35% Sensitivity: 88.2% Specificity: 91.8%

CRP = C-reactive protein, GFR = glomerular filtration rate, Cr = creatinine, Hct = hematocrit, WBC = white blood count, BUN = blood urea nitrogen, MCV = mean corpuscular volume, SBP = systolic blood pressure, MCHC = mean corpuscular hemoglobin concentration, RRT = renal replacement therapy, SAPS = simplified acute physiology score, Hb = haemoglobin, AMI = acute myocardial infarction, CHF = congestive heart failure, CABG = coronary artery bypass grafting, SOFA = sequential organ failure assessment, BMI = body mass index, LOS = length of stay, CVD = cardio vascular disorder, FEV1 = forced expiratory volume in 1 second, PAH = pulmonary arterial hypertension, RVD = right ventricular diameter, ACEi = angiotensin converting enzyme inhibitor, ARB = angiotensin receptor blocker, sPESI = simplified pulmonary embolism severity index.

**Table 3:** Study Quality as Assessed by The Newcastle-Ottawa Scale.

Study	Selection				Comparability	Outcome		
	Representativenessof exposed	Selection of non-exposed	Ascertainment of exposure	Outcome of interestwas not present at start of study		Assessment of outcome	Duration of follow-up	Adequacy of follow-up
**General critically ill patients**								
Wang et al.[9]		*		*	**			*
Bazick et al.[2]	*	*	*	*	**	*	*	*
Hunziker et al.[11]	*	*	*	*	**	*		*
Meynaaret al.[1]	*	*	*	*	**	*		*
Zhang et al.[10]	*	*	*	*	**	*		*
Hatice et al.[38]	*	*	*	*	**	*		*
**Patients with inflammation**								
Sadaka et al.[29]	*	*	*	*	**	*		*
Jo et al.[25]	*	*	*	*	**	*	*	*
Tian et al.[23]	*	*		*	**		*	*
Chen et al.[24]	*	*		*	**		*	*
Lee et al.[27]	*	*	*	*	**	*	*	*
Braun et al.[28]	*	*	*	*	**	*	*	*
Ku et al.[31]	*	*		*	**		*	*
Seyhan et al.[32]	*	*		*	*		*	*
Guray et al.[33]	*	*	*	*	**	*	*	*
**Other critically or acutely ill patients**								
Kim et al.[13]	*	*	*	*	**	*	*	*
Oh et al.[16]	*	*	*	*	**	*	*	*
Hong et al.[17]	*	*	*	*	**	*	*	*
Senol et al.[15]	*	*	*	*	**	*		*
Majercik et al.[22]	*	*	*	*	**	*	*	*
Garbharran et al.[21]	*	*	*	*	**	*	*	*
Bilgic et al.[20]	*	*	*	*	**	*		*
Kang et al.[14]	*	*	*	*	**	*	*	*
Zorlu et al.[18]	*	*	*	*	**	*		*
Sen et al.[19]	*	*	*	*	**	*	*	*
Ozsu et al.[37]	*	*	*	*	**	*		*
Mucsi et al.[36]	*	*	*	*	**	*	*	*
Miriam et al.[40]	*	*	*	*	**	*	*	*
Yao et al.[41]	*	*	*	*	**	*	*	*
Lv et al.[42]	*	*	*	*	**	*	*	*
Dinc et al.[43]	*	*	*	*	**	*	*	*
Wang et al.[39]	*	*	*	*	**	*	*	*

*, the quality of according domain.

### Group A: Critically ill patients

Four studies reported RDW as a continuous variable in general critically ill patient [1, 9–11]. The adjusted OR were in the range of 1.04 to 1.55. And 4 studies provided the AUCs of RDW for mortality prediction and its value from 0.62 to 0.73 [9–11, 38]. Only one study [2] assessed RDW as a categorical variable and along with the increase of RDW, the mortality rate of critically ill patients would greatly increase.

### Group B: Patients with infectious diseases

RDW was reported as a continuous variable in 4 studies [23, 29, 31, 32]. The adjusted OR rose from1.12 to 1.40. RDW was reported as a binary variable in 2 studies [28, 33], and we found that the increased RDW levels were associated with a higher risk of death (OR, 2.12; 95%CI, 1.82–2.46) with low heterogeneity (*I*^*2*^ = 0, *P* = 0.49). And 4 studies provided data on AUC from 0.63 to 0.76 of RDW in prediction mortality [23, 25, 31, 33]. Finally, three studies reported RDW as a categorical variable [25, 27, 29], and a rank correlation was found between the RDW levels and mortality in these patients.

### Group C: Other conditions

In this group, there was one study was obtained respectively focusing on out-of-hospital cardiac arrest victims, patients with AKI who were treated with CRRT, patients with acute dyspnea who visited the emergency department, patients after organophosphate insecticide poisoning, prevalent kidney transplant recipients, and patients in the adult internal medicine ward. In addition, 3 studies focusing on patients with acute pancreatitis, 3 studies focusing on adult trauma patients, 2 studies focusing on patients with acute mesenteric ischemia, and 3 studies focusing on patients with acute pulmonary embolism were included. Nine studies reported RDW as a continuous variable [16, 17, 21, 22, 36, 37, 40, 43], and the adjusted OR rose from 1.06 to 1.60. Six studies reported RDW as a binary variable [14, 16, 18, 19, 37, 40] and the adjusted OR rose from 1.21 to 15.47. Ten studies provided data on AUC [14–20, 37–39, 41], and its value between 0.61 and 0.82. Finally, four studies reported RDW as a categorical variable [13, 17, 22, 42]. We also found the RDW levels were significantly associated with morality with a rank correlation.

### Publication bias

The quantity of included studies in each subgroup was insufficient to evaluate publication bias. However, as the populations in our study were determined by two senior doctors, and several studies were excluded due to insufficient data available, therefore, we could not rule out the possibility of the existence of publication bias, although publication bias was considered to be absent by Egger’s test (P>0.05).

## Discussion

This systematic review attempted to quantify the association between RDW and mortality in non-cardiovascular critically or acutely ill conditions. The results indicated that in each group (general critically ill patients, patients with inflammation and other critically or acutely ill patients), no matter as a continuous variable or binary variable or categorical variable, higher RDW levels were associated with a higher mortality rate. To our best knowledge, this systematic review firstly explores the association between RDW and mortality in non-cardiovascular conditions.

Early diagnosis and timely risk stratification are particularly important to critically or acutely ill patients. It is helpful in allocation of limited ICU (intensive care unit) resources (especially in developing countries, such as China) [44], judging the severity of critical illnesses, deciding curative effects, comparing performance between different centers, and clinical decision-making [9, 10]. Physicians in the emergency department also need a simple and accurate tool to identify the patients who will benefit from hospitalization, especially those who should be admitted to ICU. In addition, an accurate prognostic model may be used as a quality improve tool through the construct of observed/ expected ratio. In recent years, a great deal of studies have attempted to find new prognostic factors [45, 46], however, due to various reasons (not readily available, expensive etc.), no predictor was widely acceptable by clinicians.

RDW is a coefficient of variation of circulating red blood cells (RBC) and is a part of the complete cell count (CBC) panel. Increased RDW reflects greater heterogeneity in red cell volume. Disorders related to ineffective RBC production (e.g. iron deficiency anemia, vitaminB12 and folic acid deficiency, bone marrow suppression and hemoglobinopathies) or increased RBC destruction (hemolysis) or blood transfusion will cause higher RDW [2]. In addition, the change of RDW is affected by many factors, such as liver or renal dysfunction, malnutrition, cancer, thyroid disease, acute or chronic inflammatory response, use of some medications, renin-angiotensin system activation, and ethnicity [1, 2, 11, 47, 48]. Until now, RDW is mainly used for the differential diagnosis of anemia (especially iron-deficiency anemia). If the possibility of anemia has been ruled out, the RDW is often completely overlooked by clinicians. However, Felker et al. in 2007 and subsequent authors have demonstrated RDW is one of the strongest independent predictors of morbidity and mortality in various cardiovascular conditions including coronary heart disease, pulmonary hypertension, acute heart failure, peripheral artery disease, stroke, or pulmonary embolism and is independent of hemoglobin level [3–8]. In addition, recent studies also found that RDW is also associated with mortality in non-cardiovascular conditions, especially in critically or acutely patients [9–33]. And RDW is not only associated with short- and long- term mortality [2], but also is associated with the incidence of bloodstream infection [2], the length of hospital stay [9, 30], the incidence of recurrent hospital admissions [33], and the use of vasopressors [25].

Although the association between RDW and mortality was reported repeatedly, the exact mechanism underling the RDW-mortality association remains unclear. However, several reasons were proposed in previous reports. Firstly, bone marrow function and iron metabolism are always influenced by systemic inflammation response [49, 50]. Meanwhile, the erythropoietin-induced erythrocyte maturation and proliferation are restrained by pro-inflammatory cytokines [51]. Additionally, pro-inflammatory cytokines usually down-regulate the expression of erythropoietin receptor which is important in the process of erythrocyte maturation [51]. This will result in the release of larger, immature reticulocytes into the circulation. It has been reported that RDW is significantly influenced by inflammatory biomarker like C-reactive protein, erythrocyte sedimentation rate (ESR), white blood count (WBC), and IL-6 [16, 23, 52, 53]. Another explanation for the RDW-mortality relationship may be oxidative stress. Lots of reactive oxygen species generated by activated leukocytes are released in the process of acutely or critically illness. And the change of RDW could be mediated by oxidative stress through increasing the fragility of RBCs, shortening the life-span of RBCs [54]. In addition, malnutrition may be another contributing factor. Recent studies have shown that the level of total cholesterol and albumin which are markers of nutrition are significantly associated with RDW [16, 30]. Other possible factors also include renal or hepatic dysfunction, chronic hyperglycemia and so on [55]. Based on the above analysis, it is reasonable to believe that RDW is an integrative marker of multiple harmful pathologic processes. However, these factors may not completely account for the relationship between RDW and mortality, because RDW is independently associated with mortality even after adjustment for total cholesterol, albumin, and SOFA score, anemia, transfusion and so on [9–11, 16, 22, 25, 29]. As for anemia, the relationship between RDW and mortality was reported to be more significant in non-anemic patients, compared with anemic patients [56]. Therefore, further studies are needed to address the association between RDW and adverse outcomes.

The clinical value of RDW remains a question. Although, the adding of RDW to established prediction models such as APACHEⅡ, SOFA, SAPS significantly improves the discriminative power [1, 11, 25], the ability of RDW in distinguishing survivors from non-survivors is sub-optimal, the pooled AUC in our study ranged from 0.68 to 0.69. Secondly, Farid and his colleagues have reported that the discriminative power of RDW is better than both APACHEⅡscore and SOFA score in patients with severe sepsis and septic shock. The low cost and readily available of RDW may strengthen its value in daily practice in the future. However, the optimal combination of RDW and other parameters or predictive model remains unclear [47, 48]. Another inconclusive issue is the value of the change of RDW over time. Chan and his colleagues reported that the increase of the level of RDW from the baseline during the first 72h after hospitalization was also associated with a higher mortality rate in patients with severe sepsis and septic shock [30]. However, this relationship was not observed in another two studies [10, 31].

## Limitations

Our study was mainly limited by the heterogeneity of the included studies. The design of study, categorization of RDW and endpoints were different between studies. And all included studies were non-random, and the inherently bias was inevitable. The change of RDW was affected by many factors, despite multiple important factors affecting RDW levels were adjusted in most studies, there was the possibility that other residual confounding factors not included in the analysis. For example, in most studies, the authors did not adjust for the levels of vitamin B12 and folic acid which may significantly affect RDW levels. However, Patel et al reported a similar strong RDW-mortality association even after adjustment for these covariables [7]. The elapsed time between the blood sampling and RDW measuring was not defined in almost all studies, this time interval may significantly alter RDW levels [57]. In addition, the blood analyzer and reagents used for measurement are different between studies. Study populations varied widely. Therefore, we do not have enough studies to conduct subgroup analysis to explore the association between RDW and mortality in each kind of disease. However, the results presented in this review are a summary of the best evidence currently available in non-cardiovascular critically or acutely ill patients.

## Implications for research

1. The exact mechanism underlying this relationship;
2. Future research should focus on subgroups of patients;
3. The value of the combination of RDW and other indexes or predictive models;
4. The value of the dynamic change of RDW levels.

## Conclusion

The increased RDW is significantly associated with a higher mortality rate and is helpful for risk stratification in non-cardiovascular critically or acutely ill patients.

## Supporting Information

S1 Checklist PRISMA 2009 checklist. (DOC)

## Abbreviations

95% CI: 95% Confidence intervals
APACHE II: Acute Physiology and Chronic Health Evaluation II
SAPS: Simplified Acute Physiology Score
SOFA: Sequential Organ Failure Assessment
HR: Hazard ratio
OR: Odds ratio
RCT: Randomized controlled trial
